# Morning sleep inertia and its associated factors: Findings from a nationwide study

**DOI:** 10.1371/journal.pone.0337992

**Published:** 2026-01-02

**Authors:** Jae Rim Kim, Hyo Jin Park, Sang Min Paik, Sun-Kyu Han, Woo-Jin Lee, Jee-Eun Yoon, Daeyoung Kim, Kwang Ik Yang, Min Kyung Chu, Chang-Ho Yun

**Affiliations:** 1 Department of Neurology, Seoul National University Bundang Hospital, Seoul National University College of Medicine, Seongnam, Republic of Korea; 2 Department of Neurology, Chungnam National University Sejong Hospital, Chungnam National University School of Medicine, Sejong, Republic of Korea; 3 Department of Neurology, Soonchunhyang University Bucheon Hospital, Bucheon, Republic of Korea; 4 Department of Neurology, Chungnam National University Hospital, Chungnam National University School of Medicine, Daejeon, Republic of Korea; 5 Sleep Disorders Center, Department of Neurology, Soonchunhyang University Cheonan Hospital, Soonchunhyang University College of Medicine, Cheonan, Republic of Korea; 6 Department of Neurology, Severance Hospital, Yonsei University College of Medicine, Seoul, Republic of Korea; Sapienza University of Rome: Universita degli Studi di Roma La Sapienza, ITALY

## Abstract

**Objectives:**

Sleep inertia, a transitional state of grogginess and impaired alertness upon awakening, varies in duration and influencing factors. This study examined its characteristics and associations with sociodemographic factors, sleep patterns, and comorbidities in a Korean adult population.

**Methods:**

Data from 2,355 participants (49.2% male, aged 19–92) in the Korean Sleep Headache Study (2018) was analyzed. Morning sleep inertia was assessed via self-reported duration in minutes using the question, “How long does it typically take for you to clear your grogginess in the morning after waking from overnight sleep?” While this single-item question was appropriate for a population-based study, it may not fully capture the multidimensional nature of sleep inertia. Multiple linear regression analyses were conducted using log-transformed sleep inertia as the dependent variable.

**Results:**

The mean (SD) sleep inertia was 15.8 (12.9) minutes. Females reported 1.1 minutes longer sleep inertia than males, although no significant differences were observed across age groups. Participants with anxiety reported 14.3 minutes longer inertia than those without anxiety, showing the largest effect size (Cohen’s *d* = 1.12). Sleep inertia was negatively associated with sleep duration (beta [95% CI]: −0.05 [−0.07, −0.02]), morning chronotype (−0.16 [−0.24, −0.08]), and habitual snoring (−0.10 [−0.17, −0.02]), but positively associated with evening chronotype (0.12 [0.04, 0.19]), insomnia (0.22 [0.13, 0.30]), excessive daytime sleepiness (0.10 [0.02, 0.19]), and anxiety (0.39 [0.14, 0.63]) (*p* < 0.02 for all).

**Conclusions:**

Sleep inertia was associated with sleep duration, chronotype, sleep-related symptoms, and anxiety. These findings underscore the need for targeted interventions to alleviate the adverse impact of sleep inertia on daily functioning.

## Introduction

Sleep inertia, a transient state of grogginess and impaired alertness upon awakening, represents a transitional phase between sleep and wakefulness [1,2]. Hilditch and McHill have conceptualized it as a “third process of sleep”, which serves to buffer abrupt transitions between sleep state and wakefulness, governed by the interplay of sleep pressure and circadian rhythm [2].

The impact of sleep inertia can be assessed through both subjective and objective methods [2]. Subjective sleep inertia is typically measured using self-reported tools, such as the Karolinska Sleepiness Scale or the Sleep Inertia Questionnaire [3,4]. In contrast, objective sleep inertia is evaluated through performance-based assessments, including psychomotor vigilance test or cognitive tasks, which quantify the functional impairment following awakening [5].

While sleep inertia may contribute to sleep continuity, it can also impair cognitive functions, such as attention and decision-making, thereby potentially impairing work performance and, in severe cases, leading to accidents [6,7]. Understanding the factors influencing sleep inertia is therefore crucial for developing strategies to mitigate its effects and prevent associated risks.

Previous research suggests that sleep inertia is most severe immediately upon waking and gradually diminishes over time. For instance, Jewett et al. reported that subjective sleepiness improved significantly within the first 40 minutes after awakening and continued to decline over the next 2–4 hours [8]. Similarly, studies by Santhi et al. and Occhionero et al. observed subjective sleepiness persisted for 1–4 hours post-awakening, showing gradual improvement, whereas objective performance impairments were shorter-lasting and recovered more rapidly [9,10]. However, these findings are based on small-scale studies conducted in controlled laboratory environments, which may not fully represent the nature of sleep inertia in everyday context.

In real-world settings, the duration and factors influencing sleep inertia remain less clearly understood. A study involving Chinese students reported an average sleep inertia duration of 11.7 minutes, with age, sleep characteristics, and chronotype identified as significant contributing factors [11]. Similarly, Carciofo et al. found that morning chronotype, sleep duration and depressive symptoms were key determinants of sleep inertia duration in a non-clinical adult sample [12]. In another population-based study, depression and anxiety were associated with prolonged sleep inertia during the postpartum period, highlighting the role of mood disturbances in adult populations [13].

While these findings provide useful insights, most prior studies have focused on young adults, specific life stages, or occupational subgroups, limiting their generalizability to broader adult populations. In Korea, Kim et al. reported that sleep inertia was more pronounced among shift workers than non-shift workers, and was influenced by chronotype and depressive symptoms depending on work schedules [14]. Another study found that sleep inertia tends to shorten with increasing age [15]. However, these studies were not based on nationally representative samples and were limited to occupational or life-stage-specific subgroup, restricting their generalizability.

Furthermore, existing evidence suggests that sleep inertia may vary globally depending on biological, behavioral, and environmental factors such as cultural norms around sleep, work schedules, napping behavior, and mental health prevalence [2,16–18]. These variations highlight the importance of conducting population-specific investigations.

These findings suggest that individual- and population-level factors including age, chronotype, occupational patterns, and habitual sleep duration, may influence the characteristics of sleep inertia differently across countries and cultures. This underscores the need for nationally representative data.

To address these gaps, the present study aims to characterize subjective morning sleep inertia and investigate its associations with sociodemographic factors, sleep patterns, and comorbidities in a representative sample of the Korean adult population. By capturing real-world data across a diverse general population, this study provides novel insights into the epidemiology of sleep inertia in Korea and offers implications for broader public health strategies.

We hypothesized that sleep inertia would be influenced not only by sleep-related variables but also by comorbid conditions such as mood disorders, and that the associated patterns may differ across specific demographic and clinical subgroups.

## Methods

### Survey procedure

This study represents the second phase of a nationwide, population-based cross-sectional survey on sleep and headache conducted in Korea between October 2018 and November 2018. Participant sampling and survey administration were conducted by Gallup Korea, a professional research organization. All interviewers received standardized training prior to fieldwork, including instruction on questionnaire content, interview protocol, and respondent interaction. Quality control procedures included field supervision and centralized review of data completeness and consistency to minimize interviewer bias and ensure data reliability. In the second phase, the methods to sample, recruit, and interview the participants were identical to those in the first phase [19], except for the updated estimated total population (n = 51,817, 851) and the final sample size (n = 2,501). This sample size, drawn using proportional stratified sampling, is sufficient for general population-level inferences.

The survey encompassed the entire nation, except for Jeju-do, and employed a two-stage clustered random sampling method to ensure demographic representation across the population. Jeju-do was excluded due to logistical limitations associated with its geographic separation from the mainland. In the first stage, 15 administrative districts (do) were selected as primary sampling units. In the second stage, smaller administrative units (si, gun, and gu) were chosen using probability proportional to cluster size. The final sample included 75 si (small to medium-sized cities), 82 gun (rural areas), and 69 gu (metropolitan areas). Trained interviewers administered face-to-face interviews using a structured questionnaire.

A total of 2,501 participants (49.5% male; 19–92 years old) completed the survey. For the analysis, we excluded 56 participants with missing data and 90 who worked in shifts. A total of 2,355 individuals (49.2% male; 19–92 years old) constituted the final sample of this study.

### Ethics

Written informed consent was obtained from all participants as part of the interview process. The study protocol was approved by the institutional review boards of Seoul National University Bundang Hospital (B-1808-484-303).

### Sleep inertia

Morning sleep inertia was assessed through the question: “How long does it typically take for you to clear your grogginess in the morning after waking from overnight sleep?” Participants provided their responses in minutes, which were categorized into two groups based on the median (10 minutes); short (≤ 10 minutes) and long (> 10 minutes).

### Chronotype

The Munich Chronotype Questionnaire (MCTQ) was used to evaluate sleep patterns on workdays and free days [20]. Chronotype was determined using mid-sleep time on free days corrected for sleep debt on workdays (MSFsc) and categorized into three groups based on the quartiles, following a previous study [21]: morning type (< 2.83), intermediate type (2.83–4.29), and evening type (> 4.29).

### Sleep duration and other sleep variables

Sleep duration, calculated as a weighted average across a week, was categorized into three groups, based on previous research [22]: short (< 6 hours), intermediate (6–8 hours), and long (> 8 hours).

Sleep quality was assessed using the Pittsburgh Sleep Quality Index (PSQI), with scores > 5 indicating poor sleep quality [23]. Insomnia was assessed with the Insomnia Severity Index (ISI), and insomnia was defined as an ISI > 10 [24]. Habitual snoring was defined as snoring at least three days per week. Daytime sleepiness was assessed using the Epworth Sleepiness Scale (ESS), and excessive daytime sleepiness (EDS) was defined as an ESS > 10 [25].

### Lifestyles and comorbidities

Alcohol consumption was categorized as frequent drinking (> 3 times per week) or infrequent/non-drinking [26]. Smoking status was classified as current users or never/former users. Regular exercise was defined as engaging in vigorous workouts, intense enough to induce sweating, on at least three days per week, consistent with prior studies [27,28]. Body mass index (BMI, kg/m^2^) was calculated using self-reported height and weight and analyzed as a continuous variable.

Hypertension and diabetes mellitus were identified through self-reports during interviews, based on prior medical diagnoses. Depression was evaluated using the validated Korean version of Patient Health Questionnaire-9, which has a total score ranging from 0 to 27, with scores ≥ 10 indicating depression [29]. Anxiety was evaluated using the validated Korean version of Generalized Anxiety Disorder-7, which ranges from 0 to 21, with scores ≥ 10 indicating anxiety [30].

### Statistical analyses

Descriptive statistics were used to compare demographic and clinical features between two sleep inertia groups. Independent t-test and chi-square tests were used for continuous and categorical variables, respectively.

Multiple linear regression analysis was conducted to examine the relationships between sleep inertia, other sleep variables, and comorbidities, adjusting for sex and age. Sleep inertia was log-transformed for analysis as a continuous variable. Variance inflation factors confirmed no significant multicollinearity.

Subgroup analyses were performed by sex and chronotype to assess whether associations between sleep inertia and related factors differed across biologically and behaviorally relevant groups. These variables were selected based on prior evidence suggesting sex- and chronotype-related differences in sleep physiology and inertia responses [11,12,14].

All statistical analyses were carried out using R Studio version 4.3.1 for Windows. The moonBook, gtsummary, and ggplot2 packages were used for descriptive statistics, regression modelling, and data visualization, respectively. Statistically significance was defined as a *p*-value < 0.05. For subgroup analyses stratified by chronotype, a Bonferroni correction was applied to account for multiple comparisons, setting the significance threshold at *p*-value < 0.017 (= 0.05/3).

## Results

### Distribution of morning sleep inertia

The mean (standard deviation [SD]) duration of morning sleep inertia in the overall population was 15.8 (12.9) minutes (Table 1). Females reported significantly longer sleep inertia compared to males (mean [SD], 16.3 [13.2] vs male, 15.2 [12.7], *p* = 0.03). Sleep inertia duration did not differ significantly across age groups.

**Table 1:** Distribution of Morning sleep inertia (Mean ± SD) across groups.

	N (%)	Sleep inertia	Log scale	*p*	Effect size^†^
**Overall**	2355 (100)	15.8 ± 12.9	2.58 ± 0.72		
**Sex**				0.03	0.09
Male	1159 (49.2)	15.2 ± 12.7	2.54 ± 0.72		
Female	1196 (50.8)	16.3 ± 13.2	2.62 ± 0.72		
**Age**				0.29	0.01
19-29	404 (17.2)	17.1 ± 15.3	2.65 ± 0.70		
30-39	398 (16.9)	16.3 ± 12.6	2.61 ± 0.74		
40-49	477 (20.3)	15.2 ± 11.3	2.58 ± 0.68		
50-59	471 (20.0)	15.0 ± 10.8	2.55 ± 0.70		
60-69	319 (13.5)	15.2 ± 14.6	2.52 ± 0.76		
70-79	228 (9.7)	15.9 ± 13.9	2.56 ± 0.77		
80-92	58 (2.5)	15.5 ± 12.1	2.56 ± 0.76		
**Sleep duration**				<0.001^*^	0.03
Short (<6 h)	274 (11.6)	18.0 ± 16.5	2.64 ± 0.84		
Intermediate (6–8 h)	1659 (70.4)	15.8 ± 12.9	2.59 ± 0.71		
Long (>8 h)	422 (17.9)	14.0 ± 9.9	2.51 ± 0.66		
Chronotype				<0.001^¶^	0.02
Morning type	580 (24.6)	14.3 ± 12.1	2.46 ± 0.77		
Intermediate type	1192 (50.6)	15.5 ± 12.9	2.58 ± 0.70		
Evening type	583 (24.8)	17.7 ± 13.6	2.71 ± 0.69		
Sleep quality				0.41	0.04
Poor	460 (19.5)	16.2 ± 15.0	2.58 ± 0.76		
Good	1895 (80.5)	15.6 ± 12.4	2.58 ± 0.71		
**Snoring**				0.38	0.05
Yes	476 (20.2)	15.3 ± 16.2	2.50 ± 0.78		
No	1879 (79.8)	15.9 ± 12.0	2.60 ± 0.70		
**Insomnia**				<0.001	0.46
Yes	386 (16.4)	20.7 ± 16.7	2.82 ± 0.78		
No	1969 (83.6)	14.8 ± 11.8	2.53 ± 0.70		
**EDS**				<0.001	0.27
Yes	355 (15.1)	18.7 ± 17.9	2.70 ± 0.78		
No	2000 (84.9)	15.2 ± 11.8	2.56 ± 0.71		
**Anxiety**				<0.001	1.12
Yes	34 (1.4)	29.9 ± 21.7	3.20 ± 0.75		
No	2321 (98.6)	15.6 ± 12.7	2.57 ± 0.72		
**Depression**				<0.001	0.35
Yes	198 (8.4)	19.8 ± 15.3	2.80 ± 0.73		
No	2157 (91.6)	15.4 ± 12.6	2.56 ± 0.72		

SD, standard deviation; EDS, Excessive Daytime Sleepiness

†Cohen’s *d* was used for comparing two groups, *η²* for comparing three or more groups

*Post hoc analysis for sleep duration revealed: Short > Intermediate > Long groups

¶Post hoc analysis for chronotype revealed: Evening type > Morning = Intermediate type groups.

Morning sleep inertia showed significant associations with various sleep characteristics (*p* < 0.001 for all), although the effect sizes were small (Table 1). Participants with short sleep duration (18.0 [16.5]) reported longer sleep inertia compared to those with intermediate (15.8 [12.9]) or long sleep duration (14.0 [9.9]). Similarly, evening chronotypes experience longer sleep inertia (17.7 [13.6]) compared to morning (14.3 [12.1]) or intermediate chronotypes (15.5 [12.9]). Participants with insomnia (20.7 [16.7] vs. 14.8 [11.8]), EDS (18.7 [17.9] vs. 15.2 [11.8]), anxiety (29.9 [21.7] vs. 15.6 [12.7]), or depression (19.8 [15.3] vs 15.4 [12.6]) also reported significantly longer sleep inertia (*p* < 0.001 for all). However, no significant differences in sleep inertia were observed with respect to sleep quality or habitual snoring.

### Characteristics of the study population by sleep inertia groups

Baseline characteristics of participants, stratified by the median of sleep inertia (≤10 minutes vs. > 10 minutes), are presented in Table 2. Participants in the long sleep inertia group were younger and predominantly females. Lifestyle variables did not significantly differ between the two groups.

**Table 2:** Baseline characteristics by sleep inertia.

	Long inertia (N = 1002)	Short inertia (N = 1353)	*p*
**Sleep inertia (minutes)**	26.3 ± 13.7	7.9 ± 2.9	<0.001
**Socio-demographic**			
Age (years)	46.9 ± 16.4	48.7 ± 16.2	<0.01
Sex (Female, %)	537 (53.6)	659 (48.7)	0.02
BMI (kg/m^2^)	23.0 ± 2.7	23.1 ± 2.8	0.46
Income ≥300 KRW (%)	771 (76.9)	1017 (75.2)	0.34
Education ≥12 years (%)	850 (84.8)	1128 (83.4)	0.37
**Lifestyle**			
Alcohol (>3/week) (%)	38 (3.8)	74 (5.5)	0.07
Smoking (current) (%)	252 (25.1)	317 (23.4)	0.36
Regular exercise (≥3/week) (%)	215 (21.5)	280 (20.7)	0.69
**Sleep**			
**Duration**			
Sleep duration, hour	7.0 ± 1.1	7.2 ± 1.1	<0.001
Short (<6 hours) (%)	132 (13.2)	142 (10.5)	<0.01
Intermediate (6–8 hours) (%)	718 (71.7)	941 (69.5)	
Long (>8 hours) (%)	152 (15.2)	270 (20.0)	
**Chronotype**			
MSFsc	3.8 ± 1.2	3.4 ± 1.2	<0.001
Morning type (%)	199 (19.9)	381 (28.2)	<0.001
Intermediate type (%)	502 (50.1)	690 (51.0)	
Evening type (%)	301 (30.0)	282 (20.8)	
**Quality**			
PSQI	3.8 ± 2.5	3.8 ± 2.3	0.85
Poor (PSQI>5) (%)	199 (19.9)	261 (19.3)	0.77
**Other**			
Snoring (%)	173 (17.3)	303 (22.4)	<0.01
ISI	6.0 ± 5.0	4.3 ± 4.1	<0.001
Insomnia (ISI > 10) (%)	220 (22.0)	166 (12.3)	<0.001
ESS	7.0 ± 4.0	6.4 ± 4.0	<0.001
EDS (ESS > 10) (%)	172 (17.2)	183 (13.5)	0.02
**Comorbidities**			
Hypertension (%)	180 (18.0)	221 (16.3)	0.33
Diabetes mellitus (%)	88 (8.8)	117 (8.6)	0.98
Anxiety (%)	28 (2.8)	6 (0.4)	<0.001
Depression (%)	111 (11.1)	87 (6.4)	<0.001

Mean ± SD for continuous variables and N (%) for categorical variables.

SD, standard deviation, BMI, Body mass index; KRW, Korean Won as a currency unit; MSFsc, Mid-Sleep on Free Days Corrected for Sleep Debt on Workdays; PSQI, Pittsburgh Sleep Quality Index; ESS, Epworth Sleepiness Scale; EDS, Excessive Daytime Sleepiness.

Significant differences were noted in most sleep variables, except for sleep quality (*p* < 0.01). Participants in the long sleep inertia group had shorter sleep durations and were more frequently classified as short sleepers, with fewer classified as long sleepers. Chronotype differences were significant, with a higher proportion of evening types and a lower proportion of morning types in the long sleep inertia group. Additionally, this group reported higher rates of insomnia and EDS but less frequent snoring.

While physical illnesses were not more prevalent in the long sleep inertia group, mental illnesses were significantly more frequent (*p* < 0.001).

### Association between sleep inertia and other sleep and mood variables

In multiple linear regression analyses adjusted for sex and age, sleep inertia (log-transformed) was assessed as a continuous variable. Short sleep duration, morning chronotype, and habitual snoring were negatively associated with sleep inertia, while evening chronotype, insomnia, EDS, and anxiety were positively associated (Table 3).

**Table 3:** Multiple linear regression analysis of sleep inertia stratified by sex.

	Total (N = 2355)			Male (N = 1159)			Female (N = 1196)		
	Beta	95% CI	*p*	Beta	95% CI	*p*	Beta	95% CI	*p*
**Sleep duration**	−0.05	−0.07, −0.02	**<0.001**	−0.04	−0.08, 0.00	**0.04**	−0.05	−0.09, −0.01	**<0.01**
**Chronotype**			**<0.001**			**<0.001**			**<0.01**
Intermediate type	ref	ref	ref	ref	ref	ref	ref	ref	
Morning type	−0.16	−0.24, −0.08		−0.18	−0.29, −0.06		−0.13	−0.24, −0.03	
Evening type	0.12	0.04, 0.19		0.11	0.00, 0.21		0.12	0.01, 0.23	
**Snoring**	−0.10	−0.17, −0.02	**<0.01**	−0.03	−0.12, 0.06	0.50	−0.21	−0.34, −0.09	**<0.001**
**Insomnia**	0.22	0.13, 0.30	**<0.001**	0.23	0.10, 0.35	**<0.001**	0.20	0.08, 0.31	**<0.001**
**EDS**	0.10	0.02, 0.19	**0.02**	−0.05	−0.17, 0.08	0.40	0.22	0.11, 0.33	**<0.001**
**Anxiety**	0.39	0.14, 0.63	**<0.01**	0.59	0.25, 0.93	**<0.001**	0.18	−0.18, 0.54	0.30
**Depression**	0.06	−0.05, 0.17	0.30	0.06	−0.11, 0.23	0.50	0.06	−0.09, 0.21	0.40

CI, Confidence Interval; EDS, Excessive Daytime Sleepiness

Sleep inertia was log-transformed and analyzed as a continuous variable.

Models were adjusted for sex and age.

Sex-specific models revealed that sleep duration, chronotype, and insomnia remained significant in both males and females (Table 3). Anxiety was significantly associated with sleep inertia only in males, while snoring and EDS was significant only in females. Depression showed no significant association with sleep inertia in any model.

**Fig 1:**
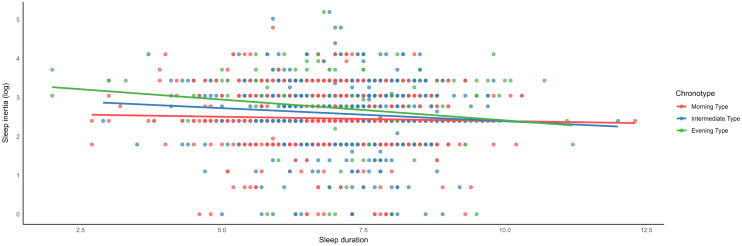
Association between Sleep duration and Sleep inertia by Chronotype.

Further subgroup analyses by chronotype revealed distinct patterns (Table 4). Insomnia was consistently associated with longer sleep inertia across all chronotype groups. Sleep duration was negatively associated with sleep inertia in intermediate and evening type (Fig 1).

**Table 4:** Multiple linear regression analysis of sleep inertia stratified by chronotype.

	Morning type (N = 580)			Intermediate type (N = 1192)			Evening type (N = 583)		
	Beta	95% CI	*p*	Beta	95% CI	*p*	Beta	95% CI	*p*
**Sleep duration**	0.00	−0.05, 0.05	>0.90	−0.06	−0.10, −0.02	**<0.01**	−0.08	−0.13, −0.03	**<0.01**
**Snoring**	−0.12	−0.26, 0.02	0.09	−0.04	−0.15, 0.07	0.50	−0.16	−0.30, −0.01	**0.04**
**Insomnia**	0.24	0.08, 0.40	**<0.01**	0.16	0.03, 0.29	**0.01**	0.26	0.11, 0.42	**<0.001**
**EDS**	0.12	−0.03, 0.27	0.11	0.01	−0.11, 0.14	0.80	0.23	0.06, 0.41	**<0.01**
**Anxiety**	1.10	0.43, 1.7	**<0.001**	0.18	−0.25, 0.60	0.40	0.25	−0.10, 0.60	0.20
**Depression**	0.14	−0.08, 0.36	0.20	0.05	−0.12, 0.22	0.60	−0.03	−0.23, 0.17	0.80

CI, Confidence Interval; EDS, Excessive Daytime Sleepiness.

Sleep inertia was log-transformed and analyzed as a continuous variable.

Models were adjusted for sex and age.

P-value less than 0.017 was statistically significant after Bonferroni’s correction.

Snoring was negatively associated with sleep inertia in evening type. EDS and anxiety were positively associated with sleep inertia in evening and morning types, respectively. Depression was not significantly associated with sleep inertia across any chronotype subgroup.

**Fig 2:**
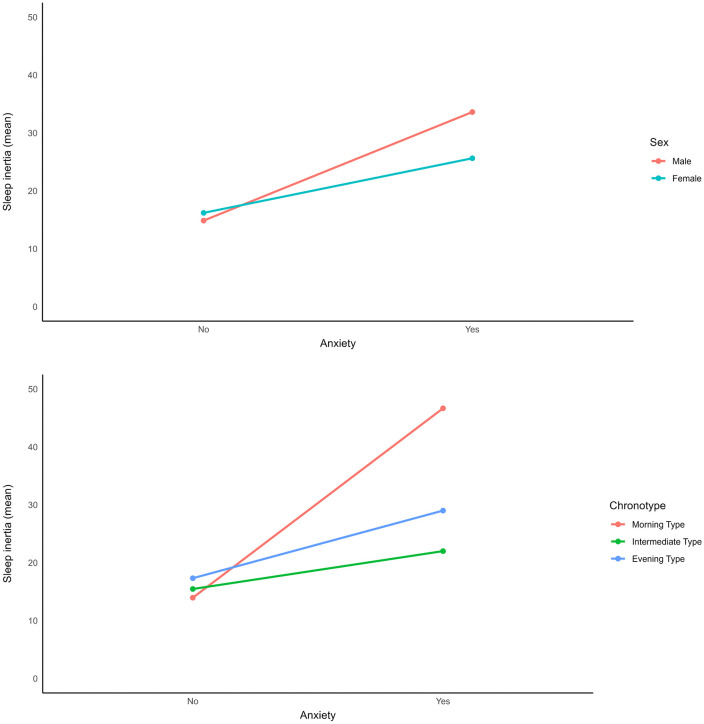
Effect of Anxiety on Sleep Inertia by (A) Sex and (B) Chronotype.

Anxiety showed the strongest association with sleep inertia, particularly in males and individuals with morning chronotype (Figs 2A and B).

## Discussion

This study is the first to investigate the distribution of sleep inertia and identify factors associated with it in a general adult population. We found that several variables were independently associated with morning sleep inertia. Specifically, sleep inertia was positively associated with evening chronotype, insomnia, EDS, and anxiety, while it was negatively associated with sleep duration, morning chronotype, and snoring, after adjusting for sex and age. Among these, anxiety exhibited the strongest association, particularly in males and individuals with morning chronotype.

The mean (SD) duration of sleep inertia in our study population was 15.8 (12.9) minutes. While sleep inertia is generally described as lasting less than 30 minutes, most previous studies have been conducted under controlled laboratory settings, often involving sleep restriction [2,6,8,9,31,32]. A study among Chinese young adults, mostly aged 19–22 years, reported a shorter mean sleep inertia duration of 11.7 (13.72) minutes, compared to our subgroup aged 19–29 years (17.1 [15.3] minutes) [11]. Although our study did not show significant differences in sleep inertia across age groups overall, a more detailed analysis revealed that sleep inertia tended to decrease up to the age of 40, after which it remained stable. These findings align with previous studies indicating that younger age is a risk factor for prolonged sleep inertia [11,15,31]. This trend may be explained by the natural age-related shift toward a morning chronotype, which stabilizes after the 40s [33]. Consistent with this explanation, we observed significantly longer sleep inertia among individuals with evening chronotype compared to those with morning or intermediate chronotypes.

Our results demonstrated that both sleep duration and chronotype were significantly associated with sleep inertia. Sleep duration was negatively associated with sleep inertia, aligning with previous studies [11]. Sleep inertia assessed through objective cognitive testing has also been shown to worse in individuals with chronic sleep insufficiency, sleeping less than 6 hours, with symptoms persisting throughout the day [34]. This association may be explained by the insufficient dissipation of homeostatic sleep load accumulated in individuals experiencing partial sleep restriction, which raises the likelihood of waking from deep sleep, thereby exacerbating morning sleep inertia [35]. Chronotype also emerged as a key factor. Morning chronotype was negatively associated with sleep inertia, whereas evening chronotype was positively associated. These findings underscore the interplay of circadian rhythm and homeostatic drives in inertia, which reflects a transitional state between sleep and wakefulness [2]. Interestingly, sleep duration was not associated with sleep inertia in morning type but was negatively associated in intermediate and evening types. This aligns with previous finding indicating worse sleep inertia in individuals with chronic sleep sufficiency, particularly during the peak of the circadian drive [32]. These results suggest that an advanced circadian rhythm may mitigate the effects of insufficient sleep on morning sleep inertia, potentially due to the nadir of the circadian drive in the morning. Conversely, normal and delayed circadian rhythms may lack these compensatory benefits, leaving them more vulnerable to the effects of sleep duration on sleep inertia [36].

Subjective sleep problems such as insomnia and EDS were also significantly associated with sleep inertia. Consistent with our findings, a population-based study in the United States reported that insomnia and EDS were linked to severe sleep inertia [37]. Insomnia management programs, such as the “RISE-UP” routine used in cognitive-behavioral therapy, have been shown to improve sleep inertia [38]. Interestingly, while previous studies have suggested a proportional relationship between EDS and sleep inertia, our findings indicate that this association is specific to individuals with an evening chronotype [11,12,39]. This suggests that EDS and excessive sleep inertia may present independent symptoms, requiring separate clinical assessments. Additionally, sleep quality was not associated with sleep inertia in our study population. While some studies have reported positive associations between sleep quality and sleep inertia [12], others, such as those employing Sleep Inertia Questionnaire, found no correlation, suggesting independence between these variables [3,11]. This discrepancy highlights the need for further research into the relationship between sleep inertia and sleep quality.

Our study also revealed that while both depression and anxiety were more common in the long sleep inertia group, only anxiety remained significant in multivariable analyses. Anxiety demonstrated the strongest association with sleep inertia, particularly among males and individuals with morning chronotype. Although no prior studies have directly examined the relationship between anxiety and sleep inertia, research has shown associations between anxiety and daytime sleepiness, particularly in morning types, consistent with our findings [40]. This underscores the importance of addressing anxiety in managing sleep inertia, especially in these subgroups.

Recently, countermeasures such as caffeine, light exposure, and auditory stimuli have been proposed to reduce sleep inertia; however, these studies were conducted in small samples under laboratory settings [41]. Our findings provided new insights into the complex relationships between sleep inertia and various factors, stratified by sex and chronotype, in a large population-based sample. Targeted interventions may be useful in addressing morning sleep inertia. For example, ensuring sleep duration may be crucial for individuals with intermediate and evening chronotypes, while managing anxiety symptoms could be beneficial for males and morning types. Additionally, insomnia symptoms should be evaluated and managed in all individuals reporting excessive sleep inertia, regardless of sex and chronotype.

This study has several strengths. It included a large, nationally representative sample of the Korean general population, spanning a wide age range, and is the first study to comprehensively investigate the relationship between sleep inertia and various factors in this population. However, several limitations should be acknowledged. First, sleep inertia was assessed using a single subjective question, which, while suitable for a large-scale survey, may lack precision. Second, the cross-sectional design precludes the establishment of causal relationships. Third, while some subgroup differences in sleep inertia were statistically significant, the effect sizes such as Cohen’s *d* and η² were relatively small, particularly for variables like sleep duration. Still, sleep duration is a well-known and modifiable factor, and the consistency of its association with sleep inertia across multiple subgroups strengthens its potential public health relevance. This highlights that even small effect sizes can be meaningful, especially when the exposure is common, and underscore the importance of considering both statistical significance and practical implications when interpreting findings from population-based studies. Fourth, the response rate for the survey was not provided by Gallup Korea, limiting our ability to assess non-response bias. Although proportional stratified sampling and trained interviewers were used to enhance representativeness, the absence of response rate data remains a methodological limitation that should be considered when interpreting the results. Fifth, the use of self-reported data for BMI and comorbidities such as hypertension and diabetes mellitus may have introduced recall or reporting bias. However, as these variables were not significantly associated with sleep inertia in our analyses, the potential impact of this bias on the findings is likely limited. Sixth, we did not conduct formal checks for regression assumptions such as residual normality or homoscedasticity, which may affect model precision. However, the consistency of findings across models and the large sample size lends some robustness to our results. Finally, we could not exclude participants with undiagnosed sleep disorders due to the absence of polysomnography or objective sleep measures. Future research using longitudinal designs and objective measurements is warranted to confirm and expand upon our findings.

In conclusion, this study provides novel insights into the prevalence and associated factors of sleep inertia in a nationally representative sample of Korean adults. We found that sleep inertia was significantly associated with modifiable factors, including sleep duration and anxiety symptoms. Anxiety emerged as the most prominent correlate, particularly in males and individuals with morning chronotypes. These findings emphasize the complex interplay between sleep inertia, mood and circadian factors. Targeted interventions, such as mood regulation strategies and circadian-based approach, may help mitigate the impact of sleep inertia and improve daily functioning at the population level.
